# Implementation of eHealth Technology in Community Health Care: the complexity of stakeholder involvement

**DOI:** 10.1186/s12913-020-05287-2

**Published:** 2020-05-11

**Authors:** Etty R. Nilsen, Karen Stendal, Monika K. Gullslett

**Affiliations:** University of South-Eastern Norway (USN), Post Office Box 235, 3603 Kongsberg, Norway

**Keywords:** Stakeholders, eHealth technology, Implementation, Primary health care, Stakeholder integration

## Abstract

**Background:**

The implementation of any technology in community health care is seen as a challenge. Similarly, the implementation of eHealth technology also has challenges, and many initiatives never fully reach their potential. In addition, the complexity of stakeholders complicates the situation further, since some are unused to cooperating and the form of cooperation is new. The paper’s aim is to give an overview of the stakeholders and the relationships and dependencies between them, with the goal of contributing this knowledge to future similar projects in a field seeing rapid development.

**Methods:**

In this longitudinal qualitative and interpretive study involving eight municipalities in Norway, we analysed how eHealth initiatives have proven difficult due to the complexity and lack of involvement and integration from stakeholders. As part of a larger project, this study draws on data from 20 interviews with employees on multiple levels, specifically, project managers and middle managers; healthcare providers and next of kin; and technology vendors and representatives of the municipal IT support services.

**Results:**

We identified the stakeholders involved in the implementation of eHealth community health care in the municipalities, then described and discussed the relationships among them. The identification of the various stakeholders illustrates the complexity of innovative implementation projects within the health care domain—in particular, community health care. Furthermore, we categorised the stakeholders along two dimensions (external–internal) and their degree of integration (core stakeholders, support stakeholders and peripheral stakeholders).

**Conclusions:**

Study findings deepen theoretical knowledge concerning stakeholders in eHealth technology implementation initiatives. Findings show that the number of stakeholders is high, and illustrate the complexity of stakeholders’ integration. Moreover, stakeholder integration in public community health care differs from a classical industrial stakeholder map in that the municipality is not just one stakeholder, but is instead comprised of many. These stakeholders are internal to the municipality but external to the focal actor, and this complicating factor influences their integration. Our findings also contribute to practice by highlighting how projects within the health care domain should identify and involve these stakeholders at an early stage. We also offer a model for use in this context.

## Background

Health care services face vast challenges that will increase in the years to come. This is due to demographic changes, such as an ageing population [1, 2] and a lack of labour. Among the Norwegian government’s main objectives and key priorities, eHealth (or digital health care) is viewed as an important way to meet these challenges [3]. Internet and digital technologies change our world, but old boundaries, thought patterns and regulations prevent Norway from fully utilising digital capabilities. Existing barriers, for example organisational routines, cause citizens to miss out on goods and services [1, 4, 5].

Alongside numerous governmental agencies around the world, the research community has also begun devoting their effort to eHealth-related topics (e.g. technology in health care, welfare technology, mobile health care and innovation of health care solutions) [6–9]. Although the digitalisation of health care services is often the focus of these projects, the complexity of eHealth initiatives often lead to failure or less-than-optimal solutions [10, 11]. The digitalisation of health care services and the large-scale implementation of eHealth is a slow process, and many initiated projects are never fully realised as part of the normal routine [12]. Although the implementation of technology in health care faces challenges similar to those of other industries, the field of health care has many (and varied) stakeholders. In the municipal domain, for instance, a multitude of stakeholders are involved—from local politicians to health care employees, and from both public and private sectors. Comprehensive studies of these stakeholders and how they act in relation to each other are needed [13–15], particularly in the context of community health care services.

The existing research on this topic largely focuses on hospitals as the empirical arena (see, for instance, [13, 16–18]). However, while a hospital is essentially one organisation, often with few geographically proximate buildings, a municipality consists of several embedded and loosely affiliated organisations. Hence, the municipal health care context is highly complex with regards to number and character of stakeholders.

Research underscores that collaboration among these stakeholders can facilitate eHealth implementation processes [19, 20]. This is due not only to the infrastructure and organisation of services, but also to differences in the needs of the patient and other users. Services on the specialist level (i.e. the hospital) often consist of the short-term delivery of health care to patients in one physical location, while municipalities are responsible for providing long-term services to inhabitants with complex health care needs—both in institutions and, increasingly, in their own homes. The specific long-term care service explored in this research project is the use of eHealth in nursing home care, where the end users are mostly people diagnosed with dementia. These end users are not active users of eHealth technology due to their cognitive impairment.

Municipalities have assumptions about which stakeholders are relevant at the outset of innovative projects like the implementation of eHealth technology in community health care services. These assumptions are based on experience with implementation projects, but it is an inherent characteristic of innovative projects that the map cannot be drawn based on experience alone. Empirical mapping has shown that a number of stakeholders emerge during the implementation. In light of this, we aim to answer the following research questions: *Who are the stakeholders in the implementation of eHealth technology in municipal health care, and what is the nature of the relationships among them?*

To answer these questions, we conducted a qualitative study as part of a larger project in Norway. Norwegian health care services are predominantly delivered by the public sector. The use of eHealth has not realised its full potential in this setting, and there is a lack of studies on the implementation of simple technology in this context. The remainder of this paper is structured as follows: First, we present the field of eHealth and stakeholder theory; next, we present the research method and the case (i.e. The Digital Surveillance project); we then present the study findings, after which we discuss them and our contribution to both theory and practice; and finally, we conclude our study.

### eHealth

eHealth is a fairly young field of research [21], one that is generally cross-disciplinary. The term ‘eHealth’ can be defined as: ‘The application of information, communication, computing, and sensing technologies across the entire range of functions and processes constituting the practice and delivery of health care services’ [21]. Following this definition, it is clear that successful eHealth implementation involves much more than just technology [22–24] and includes stakeholders and processes across a broad range of functions in the health care field [23]. eHealth is enabled through integrated applications in the healthcare environment, and includes technologies related to computing, communication and sensing [21].

A study conducted in the Netherlands concluded that organisational readiness is an important factor when implementing and adopting eHealth initiatives [25]. The authors define ‘organisational readiness’ as ‘the availability of the needed organisational resources for adoption’ [25], but as their study was conducted in hospitals, the operationalisation of this concept must be adapted to the context of community health care in municipalities. To be ‘organisationally ready’, the organisation must be able to adapt to the change process and deal with the intended and unintended consequences of that change—salient factors in implementing and adopting eHealth technology [25].

Previous research in eHealth technology implementation in a municipal setting has identified a lack of tools for evaluating the technology implemented. Moreover, eHealth technology is seen as the end goal, rather than a means to improve services in nursing homes. These elements are seen as the main risks in an implementation process [26], which suggests there is something of a social experiment when implementing eHealth technology in real-world settings, especially in municipal health care [26, 27]. It has been argued that eHealth initiatives will only succeed if the patient is kept at the centre and if socio-cultural/behavioural, organisational, financial, political and technical barriers are addressed with the objective of empowering patients [21, 28]. With this in mind, however, the users of eHealth technology are usually health care workers, and this may hinder eHealth technology implementation initiatives, due to lack of experience and knowledge about the technology [26].

The implementation of eHealth technology is contextually dependent, and factors that affect such contexts are organisational issues, technological infrastructure and human action [27]. The implementation of eHealth technology may also be seen as changing practice, and as a stage in the innovation of services [29]; this includes active collaboration and co-creation between vendors and consumers [30, 31]. Those who decide to implement eHealth technology into municipal health care must be positive towards the changes and the possibilities offered by the technology; however, municipal health care organisations seem to struggle with understanding these premises [32]. In the context of the present study, successful implementation of eHealth technology was therefore dependent not only on the technology in question, but also on the multiple stakeholders involved.

### Stakeholder theory

Although stakeholder theory was developed primarily for investor-owned corporations [33], several authors have found the approach useful in other contexts, such as e-government, social media, local government issues and project management (e.g. innovation projects) [18, 30, 34–36]. Within health care, the stakeholder issue is frequently an area of focus, with the patient often seen as an important stakeholder (see, for instance, [35]). In the field of eHealth, several studies have applied a stakeholder perspective, but, as noted above, most of these were performed in the context of a hospital [13, 37]. In these and other studies, the complexity of the health care field and of the hospital context has been documented.

However, as we will argue, community health care is an even more complex setting, making the implementation of eHealth technology in this context all the more complex, as well. Primary health care depends on inter- and intra-organisational cooperation between stakeholders; these stakeholders, though sometimes external, are primarily gathered under the umbrella of a municipality, and there is often an asymmetric relationship between these actors concerning power, resources and knowledge. While there are some exceptions to the overwhelming focus on hospitals (see, for instance, Schiller et al. [37]), literature targeting eHealth is lacking, and the mapping lacks depth regarding stakeholders internal to the municipality. Moreover, to our knowledge, the stakeholder perspective has not been extensively applied to eHealth technology implementation in the context of community health care services.

In our stakeholder mapping, we base our concept of the ‘focal actor’ around Freeman’s [38, 39] seminal work. Although the focal actor in the context of implementing eHealth technology in community health care bears little resemblance to a powerful focal actor in an industrial context, it is where the actual implementation takes place. In a complex implementation project like the one under study, the focal actor (e.g. the health care institution) has relationships with several stakeholders, which complicates the management of the implementation. Additionally, stakeholders have relationships, and sometimes, these relationships are independent of the focal actor. These relationships can be labelled as ‘stakeholder involvement’ [40] and ‘stakeholder integration’ [30], and are partly overlapping.

Although previous research has considered stakeholders as part of the process, there has been less focus on the nature of the involvement and the extent to which they should be involved [41]. Stakeholder involvement might vary in intensity and indeed has been described as occurring on a continuum, from passive to more active [40]. Passive involvement is operationalised as simply sharing information, while active involvement—which is based on the logic of private or social enterprises—is operationalised as ‘stakeholder representation’ [40]. Stakeholder representation (e.g. representation on a board) may be compared to the steering committee of a project in the public sector, such as the one in our study. Though this is a simplification, as not all stakeholders in the context of technology implementation would have representatives on a steering committee, the continuum is a useful concept in the context of municipal health care.

Stakeholder integration can be seen as including both the mapping and the management of stakeholders, regarding which—and to what extent—stakeholders will be integrated [30]. In their study exploring the integration of internal and external stakeholders in the health care industry, Jonas and Roth [30] categorised stakeholders from the vendor’s perspective in a supplier−customer relationship. The authors differentiated between internal and external stakeholders, and defined four modes of stakeholder integration: passive, reactive, mutually integrated and pro-active. This represents a kind of continuum, moving from stakeholders occupying a distant role (for example, in the test purchase of technology); to acting as a source of information for each other; to acting as partners; and, finally, to being pro-active in the co-creation process. The authors see the developer/vendor as the focal actor—in the present study, though we see the health care institution as the focal actor (i.e. the customer), we nevertheless find the framework useful for the categorisation of stakeholders.

## Methods

The methodological approach used in this study is longitudinal, qualitative and interpretive. A case study design was applied as the issues under study were processes inextricably linked to their contexts. Second, the complexity of the case made the study unfit for a cross-sectional questionnaire; there were too many ‘variables’ for the number of observations made [42]. The case we studied was purposely sampled [43]. This longitudinal case study was conducted from 2013 to 2017, with several data collection points over the 4 years. The case was an innovation project, called the Digital Surveillance Project, which was financed by the Research Council of Norway’s regional research funds (project no. 234978).

### Case—the digital surveillance project

As part of the Digital Surveillance Project, eight municipalities collaborated as a network with two technology vendors to develop and implement sensors and digital communication in local nursing homes and the home nursing service. The project was originally initiated by vendors looking for an arena in which to test and co-develop their products. They approached the municipalities and one of the universities and funding was consequently secured. The project continued in a ‘triple-helix’ fashion [44], whereby each party found that they had mutual interest in the implementation of eHealth technology.

The planning of the implementation differed from municipality to municipality. In some municipalities, it was a top-down process initiated by the politicians with the goal of saving money; in others, it took the form of a bottom-up project initiated by the municipal administration or project managers [19]. The municipalities defined which area to begin with, ultimately deciding on night-time surveillance technology for patients with dementia, so-called ‘night wanderers’. The implemented technology included sensors on doors and beds and electronic bed mats for use at night. A web portal facilitated communication through computers and mobile units. Most of the participating municipalities already had some form of eHealth technology installed—for example, alarm systems. With the new elements of these systems, sensor technology was closely tied to a web portal that could support multiple technologies in various categories. Each patient could receive services tailored to their individual needs, and any changes in those services, based on time of day or changes in diagnosis, would happen through the web portal. When an incident occurred, an alarm would appear in the portal, and the system was programmed to send a corresponding alarm to the nursing staff’s mobile unit or computer. After the staff checked on the patient, they could sign for the alarm in the system.

The municipalities implemented surveillance technology supplied by vendors (who also participated in the Digital Surveillance Project) on a small scale in nursing homes and assisted living units. The vendors installed the devices and instructed the users (employees) on how to operate them. Simultaneously, a research project followed the projects organised by the eight municipalities and vendors; the research project was a collaboration between the University of South-Eastern Norway and the University of Agder, Norway. All parties, municipalities, vendors and research institutions were represented on the steering committee for the research project, which was established after the implementation was initiated.

In addition to the implementation activities taking place in the health care institutions, health care workers, health care managers, vendors and researchers participated in seven workshops over 3 years. The purpose of these workshops was for participants to learn from each other’s experiences and to refresh their own knowledge about relevant subjects, such as service design. During this period, as our awareness of the variety of stakeholders increased, other stakeholders were invited to join the workshops (e.g. employees from several IT departments).

Although some municipalities experienced positive outcomes from the implementation of the technology, few decided to implement it on a large scale in their institutions. The pilot projects proved difficult to continue after the completion of the pilot project, and lacked the resources needed for continued implementation, as seen also in the Digital Surveillance Project.

### Data collection

Part of a larger project [19, 29], the data for this study came from 20 interviews with employees on several levels, including project managers, middle managers, health care providers, next of kin, technology vendors and representatives of the municipal IT support service. Taken together, the participants represent all eight municipalities included in the study. The interviews were conducted at various time points during the implementation process, and comprised in total 16 individual interviews and 4 group interviews (*n* = 20) (see Table 1). Seventeen of the interviews took place at the participants’ workplace, and three took place at one of the universities. These interviews, conducted with purposefully selected individuals, focused on the stakes each one had in the implementation project, as well as on their experiences.

**Table 1:** Overview of interviews

Number of interviews	Individual/group interviews	Number of informants	Informants
1	Group interview	9	Health care workers, including middle managers
1	Group interview	4	Vendors
2	Group interview	4 and 3	IT support service
5	Individual interview	5	Health care workers
2	Individual interview	2	Department managers
3	Individual interview	3	Project managers
4	Individual interview	4	Next of kin
1	Individual interview	1	Vendor
1	Individual interview	1	IT support service

Table 1 gives an overview of the interviews on which this paper is based. Some participants whom we interviewed individually also participated in the group interviews.

To complement the interview data, we drew on data from observations of meetings, trainings and workshops with participants from municipalities, vendors and researchers, conducted as part of the larger project. While these data were not collected at the time for the purposes of mapping stakeholders, they provide a useful lens through which to examine the complexity of the stakeholders.

### Data analysis

As mentioned earlier, this study was part of a larger longitudinal qualitative study in which data collection and analysis were conducted. Over time, it became apparent that the complexity of stakeholders was an important issue, which led to revisiting the data and conducting additional data analysis. The data were analysed using a coding system based on categories derived both from the data and from theoretical perspectives. Following Jonas and Roth [30], codes used in the deductive analysis identified stakeholders as internal and external, and characterised them according to their modes of integration. Further analysis was conducted inductively, once tentative findings began emerging from the data [45]. Through this analysis, we identified the centrality of each group of stakeholders in the project, labelling these positions as ‘core’, ‘support’ or ‘peripheral’.

The strength of the relationship between stakeholders was inferred from the qualitative interviews—in particular, the participants’ descriptions about the stakeholders with whom they cooperated (and how), as well as what they identified as challenges to cooperation. The researchers ensured validity through iterative, collective reflection and discussion.

#### Ethical considerations

This sub-study and the larger study of which it was a part was approved by the Norwegian Social Science Data Service (ethical approval nos. 34,831 and 36,230). Participants gave their informed consent and had the opportunity to withdraw from the study without penalty. Care was taken to ensure participants’ anonymity throughout the study.

## Results

### Identification and classification of stakeholders

When the research and innovation project was being developed, it was outlined as a triple-helix cooperative effort between municipalities, vendors and researchers. In the process of defining the initial project and research aims, a mapping of the stakeholders assumed to be involved in or affected by the implementation of eHealth technology in the context of a community health care service was undertaken. This map was based on our understanding of the empirical arena at the beginning of the project, and consisted of the following stakeholders: elderly in need (or potentially in need) of help in their own homes and in nursing homes; employees in municipal health and care services; families and next of kin of the elderly; private sector vendors of surveillance technology; governments; and the public sector, including hospitals, educational institutions and voluntary organisations. These categories were identified based on empirical observations conducted in the very early stages of the project, and mirror both the researchers’ and the practitioners’ perception of the ‘landscape’.

This initial list of stakeholders was created to guide the research team and practitioners in involving the stakeholders. In the larger study, we narrowed our scope and focused on the employees in municipal health care and the next of kin of the persons needing help. Despite the narrow focus, the study underscored the high number of stakeholders. Interestingly, the new list of stakeholders exceeded the list identified in the preliminary mapping and underestimated the seeming importance of the relationships among them. However, throughout the study, and inspired by the structured approach for identifying health care actors in eHealth adoption in hospitals [13], the categorisation and identification of stakeholders involved in this implementation evolved (as illustrated in Table 2). This was a result of our observation and continuous data analysis. The stakeholders added were health care managers, non-health care staff and IT departments. In Table 2, we list the identified stakeholders, and include a short description.

**Table 2:** The identified stakeholders

STAKEHOLDERS	DESCRIPTION
End users	Patients in nursing homes and home-care units.
Next of kin	Families and next of kin of end users
Health care staff	Staff employed in the municipal health care units
Health care Managers	Top and middle managers employed in the municipal health care units
Non-health care Staff	Employees in the health care units; cleaning staff, janitors, etc.
eHealth Project Manager	Manager overseeing the eHealth implementation project
IT department	Support staff in the municipalities and inter-municipal IT department
Vendors/Innovators	Private SMEs, and innovators of the technology
Local politicians	Politicians in the municipalities
Municipal administration	Municipal hired staff
Government	Government, Ministry of Health and Care services, Directorate of Health and the Directorate of eHealth

The stakeholders listed in Table 2 can be categorised along various veins, as suggested by Mantzana et al. [13]. As mentioned earlier, we chose to categorise the stakeholders as external and internal, meaning that the stakeholders were internal or external to the health care institution (i.e. the focal actor). This distinction was important to the mapping of the relationship between stakeholders, since we assumed that the nature of the relationships between internal stakeholders would differ from the relationships between internal and external stakeholders. Furthermore, the complexity of community health care on the municipal level called for special focus on internal stakeholders and their relationships, as the following account will show.

#### Internal stakeholders

The internal stakeholders identified in this study are the end users, health care staff, health care managers, non-health care staff and the eHealth project managers. The motivation for technology implementation varied among municipalities, but they shared a concern for end users and the goal of increasing the level of safety and quality of care. The end users in this case were predominantly patients with dementia in nursing homes and home care. Due to their illness, they were not always aware of the technology in use, nor were they in charge of the fine tuning of technology and the setting of individual parameters. Although the end user was the target of the implementation, the health care staff members were largely the actual users of the technology.

The health care staff consists of individuals who use the technology on a daily basis, and they represent the customer (the municipality) that buys and implements the technology in this project. This staff category consists mainly of two professional groups: certified nurses and nurses’ aides. They are characterised by a low level of technology proficiency and a high level of concern for the well-being of the patient (for a detailed description, see Nilsen et al. [29]). During the initial stages of the implementation process, they were trained by vendors in how to use the technology, and a group of staff members were appointed as super users.

Managers in the health care service have the general responsibility for the implementation. Simultaneously, they have overall responsibility for the safety of the patients and the staff. For the managers, this is a dilemma, due to the challenges presented by ‘technology in the making’. When the technology is not fully developed and is, at the same time, in the process of being implemented in a new context, it does not always work as intended. This poses a threat to conditions perceived as important for the safety of the patients, namely stability and predictability in the service.

Non-health care staff members (e.g. cleaning staff, janitors and kitchen staff) are also stakeholders in the implementation process, although they are rarely identified as such in the pre-implementation phase. For example, the cleaning staff inadvertently disconnected the power source for the sensors or moved the sensors into a position where they did not work, since they had not been informed about, nor been included in, the project. However, even had they been informed, they had limited awareness both regarding their inclusion and their responsibility to take the technology into account when making alterations to patients’ rooms. The following quote from one of the night nurses illustrates this: *[…*] *because they changed the room around so it became impossible to use the electronic bed mats, for example. And then they said, ‘Oh, yes—no, we did not think of that’. So, they never contacted me as a night nurse or super user nor think a bit like ‘Yes, okay, she [the patient] is going to have her room in this and this way. What do we have to consider here and now?’*

The division of internal and external stakeholders is useful due to the complex context of the municipality. However, not all stakeholders can be easily defined as either internal or external, as exemplified by the eHealth project managers in each municipality. They are responsible for the implementation project but depend on health care institutions and local middle managers for cooperation and the success of the implementation. They can be characterised as external to the health care institution in that they usually have their employment (permanent or temporary) connected to the municipal administration. At the same time, they are usually also closely tied to health care institutions due to their professional identity as former nurses or managers in the same institutions. As the following quote illustrates, project managers are an important stakeholder in the implementation project, with connections both internally and externally: *It does not matter as long as it [the technology] works. The night nurses must feel assured that if a patient falls out of the bed or leaves the bed, if they go out the door, the alarm must work.*

#### External stakeholders

By ‘external stakeholders’, we mean external to the health care institution. We identified the following external stakeholders in this implementation project: patients’ next of kin; eHealth project managers; IT departments; vendors; local politicians; municipal administration; and central government, represented by several government agencies.

The patients’ ‘next of kin’ is a group of individuals who play a varying role in terms of being stakeholders. This group is far from homogenous, and in general, they appear to be only moderately engaged in this implementation of eHealth technology.t We identified three different categories of next of kin: the knowledgeable next of kin, who may be demanding but also reluctantly await the development of the service; the caring next of kin, who can be demanding and aggressive but also arrange much on their own; and the indifferent next of kin, who often have low demands but would like to be informed. This latter category seeks little information on their own, as illustrated by the following quote: *Well, I know that some of the others [next of kin] have taken initiatives in this, but as long as Mom is doing okay, I do not feel the need for a meeting. But when they have those meetings, we usually show up – me or one of my brothers.*

IT departments, local or inter-municipal, are responsible for the hardware, software, maintenance and IT infrastructure in municipalities. Ironically, despite their key role in the technological development and the general digitalisation of municipalities, these departments become involved at a very late stage in the implementation process—or not at all, as the following quote illustrates: *[…] Of course, we should be a part of this, but we are not invited. So, nobody includes the resources that we have to offer into the equation, in terms of driving the process.* They appear to have a low degree of involvement in general [46, 47], and when they are involved, there is a high degree of hostility and resentment toward cooperating. The resentment is due to their late involvement in the project, combined with their responsibility for the safe and stable running of the information systems. The project represents a dilemma for them, since the ‘project in the making’ is incompatible with the demand for security and predictability technology [29].

The involved vendors are private SMEs (small and medium-sized enterprises), which are also the innovators of the technology utilised in the project. They are deeply involved in the implementation and cooperate with municipalities. The eHealth technology implemented in this study is not an ‘off-the-shelf product’; on the contrary, the products are under development and must be adjusted in the new and highly variable context of the municipal health care sector.

Local politicians are sometimes initiators of implementation projects with varying motivations—often economic considerations and the potential savings to which the technology can contribute. They are also motivated by the ‘modernity’ of the project. The initiation phase is usually followed by less interest in allocating resources to an implementation with a broader scope. The municipal administration is central to the decision-making process when the choice of what kind of technology to implement is made. The administration is responsible for effectuating the decisions made by local politicians and ensuring that laws and regulations are followed. In Norway, nearly 100% of community health care is public; and the central government plays a role as a stakeholder in the political advice and decrees sent to municipalities on how they should invest in eHealth technology.

### Mapping and categorising the relationships among the stakeholders

The implementation of eHealth technology is a process that involves technology, people, infrastructure and technology acceptance. The implementation under study proved, however, that this issue is far more complex than anticipated, due to the municipal context and the surprisingly high number of stakeholders. While our study identified multiple stakeholders influencing the implementation of eHealth technology in municipal health care, we found the relationships among stakeholders—or, in some cases, the lack of such relationships—particularly interesting.

**Fig. 1:**
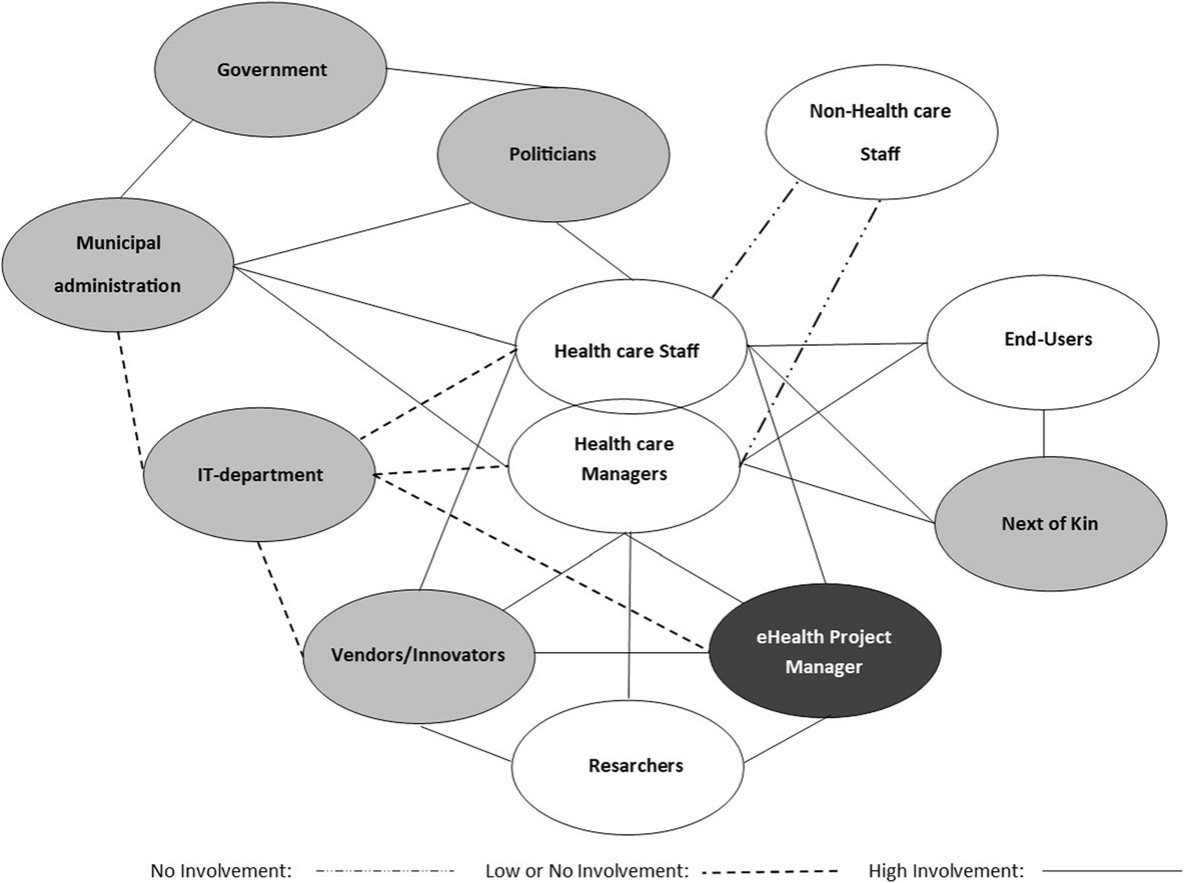
Relationships among the stakeholders in the implementation process

In Fig. 1, the lines between stakeholders indicate the relationships among them. The unbroken lines illustrate relationships between stakeholders, and the dotted lines illustrate relationships with low or no involvement with other stakeholders. This will be explained in greater detail below. In Fig. 1, the filled circles represent external stakeholders, while the white ones represent internal stakeholders. The circle for eHealth project managers is black, indicating that they are both internal and external. As can be seen in the figure, the internal stakeholders, end users, health care staff, health care managers, non-health care staff and eHealth project managers have relationships with each other and many external stakeholders. Additionally, external stakeholders have relationships that are sometimes independent of internal stakeholders; these relationships, however, are beyond the scope of this study.

As seen in Fig. 1, the researchers in this project are also considered stakeholders. They had a strong relationship with vendors, healthcare managers and the eHealth Project Manager, and most of these relationships were connected through the steering committee and other meetings. Not shown in the model is the researchers’ relationship to the other identified categories. Although we had an outside/inside function, we recognise our presence might have affected the ways in which the process was conducted.

## Discussion

Above, we have mapped the stakeholders, and then identified and categorised the relationships between them, demonstrating a complex picture. The process of mapping stakeholders followed the method suggested by Achterkamp and Vos [14], as it was emerging and ongoing throughout the implementation process, rather than taking a step-by-step approach. As mentioned above, we divided internal and external stakeholders, since the map illustrates that there are relationships within and between these two categories of stakeholders. In the following, we will discuss the nature of these relationships.

### Relationships among internal stakeholders

The health care staff and managers have relationships with other internal stakeholders: specifically, the patients (the end users), the next of kin, and the eHealth Project Manager. Their relationships with the end users, the next of kin and the eHealth project managers are illustrated with unbroken lines in Fig. 1, which indicate that the degree of involvement in these relationships is high and active [40].

The relationship between the health care staff and managers, on one hand, and the non-health care staff, on the other, is illustrated by a dotted line. The dotted lines illustrate the relationships with low-intensity involvement [39], and these relationships are characterised by non-integration or, at best, a passive or reactive mode of integration [30]. The non-health care staff is not involved in the implementation process (illustrated in Fig. 1 with uneven dots) and represents stakeholders often ignored in the early processes of the projects [48]. The lack of involvement results in contra-productive behaviour from the non-health care staff regarding the implementation of the eHealth technology. For instance, the cleaners turned the bed sensors towards the wall while they were cleaning, and as a result the sensors did not function.

### Relationships between internal and external stakeholders

While the health care staff are obviously involved with the end user when new technology and routines are introduced, the relationship with the patient’s next of kin is of particular importance since most users suffer from dementia and therefore cannot provide informed consent. Next of kin are contacted and informed through messages and meetings concerning the technology implementation.

The health care staff, including the managers and the eHealth project manager, maintain close cooperation with external vendors during the implementation, which is atypical for large purchases made by the public sector due to regulations of public procurement. In this triangle, relationships are pro-active [30], and they co-create both the technology and the health care services. These stakeholders meet at regular intervals in the workshops as part of their participation in the project, but even more importantly, they continuously meet throughout the implementation process over technological deviations (both small and large). Not all these deviations are due to the technology itself or the use of it, but are frequently due to the technological infrastructure in the municipality or the region for which the IT department is responsible.

As we see from Fig. 1, these stakeholders (the health care staff, managers, the eHealth project managers and the vendors) are connected to the IT department by dotted lines and indicate a relationship with low involvement [40] and passive or non-integration [30]. The eHealth project manager has a dual role, both as an internal and external stakeholder. The modes of the relationships that these project managers have with the health care staff, the health care managers and the vendors/innovators are mutually integrated and pro-active [30]. The relationship with the municipal IT department is, however, either low on involvement, non-existent or reactive. This mode of low involvement or reactive relationship is a common theme for how the IT department relates to the other stakeholders in most municipalities. On the IT department’s part, excessive use of technical language, lack of knowledge of the municipal health care service and their needs, new technology and lack of service orientation are identified as possible reasons. In the same vein, the relationship between the vendor/innovator and the IT department has been the source of many complications and has negatively influenced the outcome of the implementation, despite these two stakeholders using the same terminology with roots in the same epistemic cultures [49].

The relationship between the IT department and the health care institution does paint an ambiguous picture across various municipalities. A few municipalities established a relationship with the IT department, with high involvement and integration prior to the implementation, but as a rule, this relationship was established at a late stage in the implementation process. Low involvement and lack of integration between stakeholders can be seen as a barrier to successful implementation. Our findings show that not involving the IT department slows the implementation process and may create additional barriers for success. Moreover, as we found, this can sometimes result in resistant behaviour from the IT department [29].

**Fig. 2:**
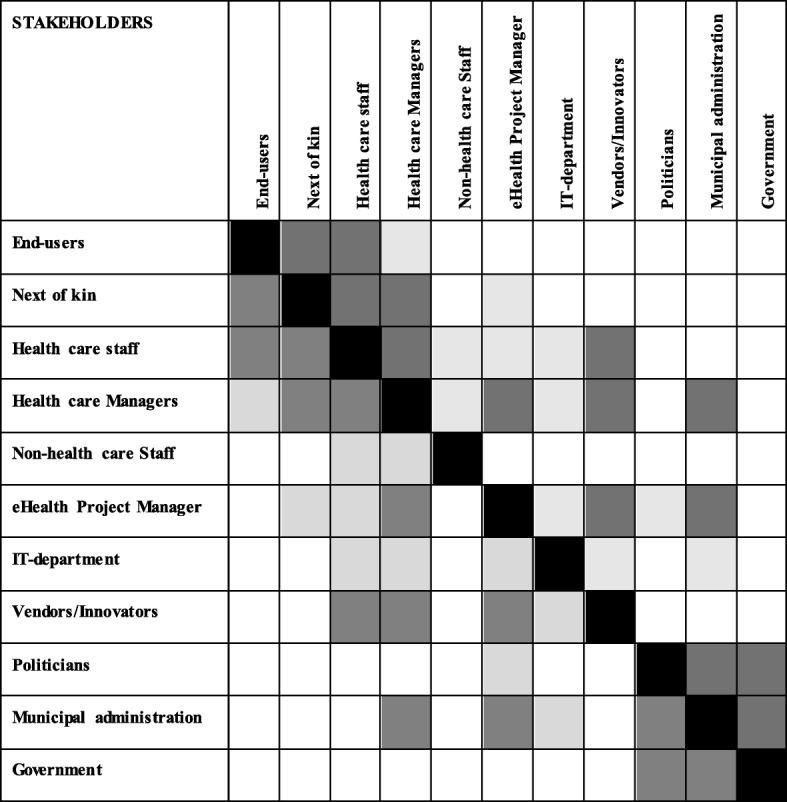
Mode of stakeholder integration

Furthermore, Fig. 2 illustrates the mode of stakeholder integration among the stakeholders that emerged in the implementation project. The empty squares illustrate that the stakeholders are non-integrated in the implementation of eHealth technology, whereas the light grey indicates a passive or reactive mode. The dark grey indicates a mutually integrated or pro-active mode. The black squares illustrate that the stakeholders have no mode towards themselves.

Here, findings show that a lack of relationships, or passive or reactive modes of relationships, create barriers for the implementation of eHealth technology. Not all relationships require pro-active or mutually integrated modes of integration, as some are more crucial than others. In general, however, we find that the importance of several stakeholders is underestimated in the implementation process. In the following, we will present a model for stakeholder integration in projects implementing eHealth technology in primary health care.

#### Model to integrate stakeholders in innovative implementation projects in primary health care

The above analysis of stakeholders and relationships offers direction for creating a model of stakeholders and their involvement in the implementation of technology in primary health care. Table 3 gives an overview of the internal and external stakeholders, and a description of their degree of involvement. Drawing on Jonas and Roth’s [30] four modes of stakeholder integration, we divide the stakeholders into three categories: core stakeholders, support stakeholders and peripheral stakeholders.

**Table 3:** Modes of identified stakeholders

Classification	Stakeholders	Internal	External	Mode of integration		
				**Pro-active**	**Mutually integrated**	**Passive/Reactive**
Core stakeholders	Health care staff	X		X		
	Health care Managers	X		X		
	eHealth Project Manager	X	X	X		
	IT department		X	X		
	Vendors/Innovators		X	X		
Support stakeholders	Non-health care Staff	X			X	
	Next of kin		X		X	
Peripheral stakeholders	Local politicians		X			X
	Municipal administration		X			X
	Government		X			X
	End users	X				X

The stakeholders in a mutually integrated or pro-active relationship—the ‘core stakeholders’—are crucial for the success of the implementation project. Without their involvement, the barriers for success will be high, and the implementation is likely to fail. Examples here are the involvement of the vendors and the IT department. The relationship with the vendors was pro-active, but the IT departments were not involved in several municipalities in the project. With their knowledge and general perspective, they should, however, have been an integrated part of the project [47].

Furthermore, the group of stakeholders we labelled ‘support stakeholders’, such as next of kin and non-health care staff, must be mutually integrated into the project to be considered partners. Without their involvement, the project can still succeed, but their involvement would smooth the path towards success. Finally, the last group of stakeholders—the ‘peripheral stakeholders’—are either passive or reactive. In this project, these are politicians (local, national and municipal administration) who contribute to the policymaking and decision-making aspects of the projects. These stakeholders can be drivers for change but are not directly involved in the implementation of projects.

**Fig. 3:**
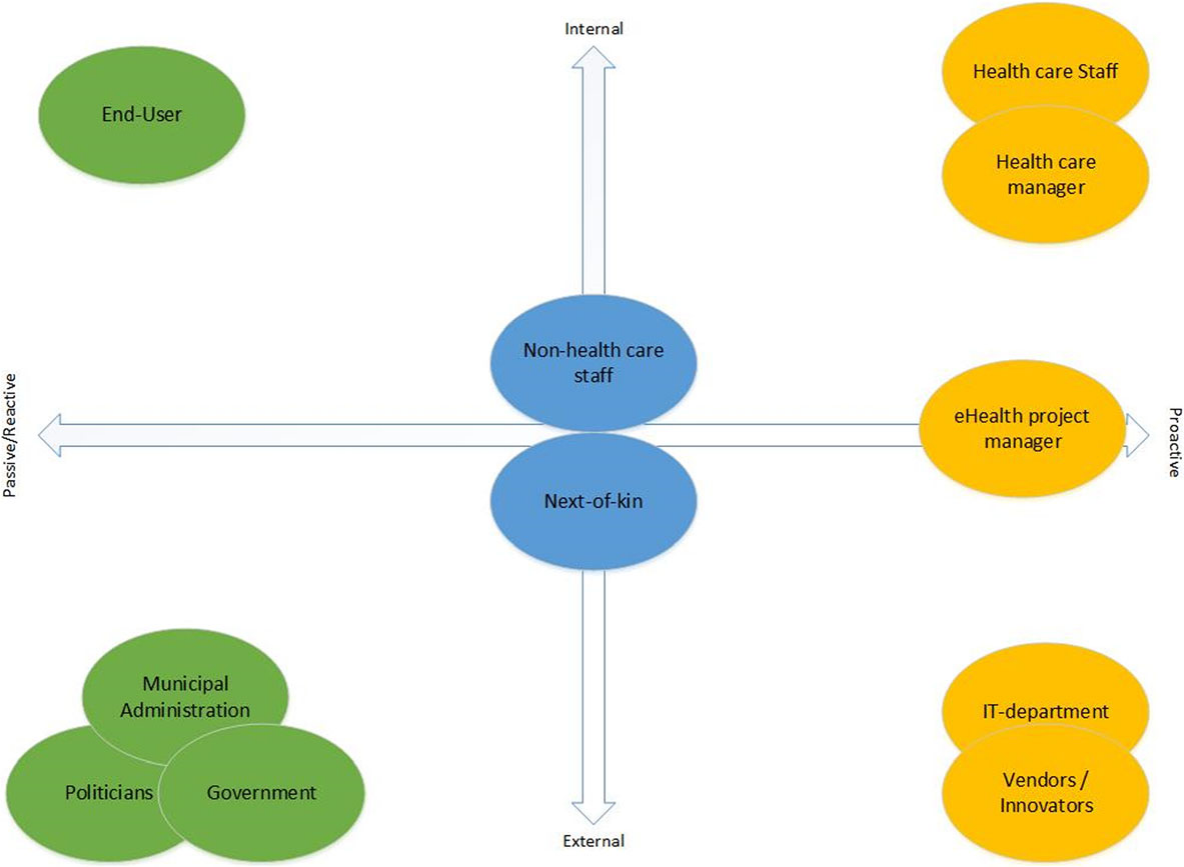
Model of stakeholders in eHealth technology implementation in community health care

Figure 3 shows a model of stakeholders illustrated along the two dimensions: 1) from internal to external; and 2) from pro-active to passive/reactive.

Together, these three categories of stakeholders contribute to the implementation of eHealth technology initiatives in municipal health care; in this study, we have identified gaps in the actual involvement and integration of all three categories of stakeholders. While we are aware that there are best practices when implementing technology in the health care sector, we also recognise the shortcomings of the actual process presented in this work. In a perfect world, all stakeholders would be identified and involved from the initiation phase of the project; however, many of the stakeholders were largely involved late in the process, while some were not included at all. An example here is the lack of involvement concerning the IT department. This skilled and highly important stakeholder was often not consulted or involved until the technology had been ordered and/or arrived on the premises, ready to be implemented. The involvement of such an important stakeholder much earlier in the process could have prevented unnecessary complications in the implementation process.

## Conclusion

Our findings show that there is a need to clarify the relationships among stakeholders and ensure good communication channels. Study findings contribute to both theory and practice by identifying multiple stakeholders in a municipal eHealth technology implementation project. In addition, we have categorised the relationship structures among the stakeholders. Finally, we have developed a model for stakeholder relationships by classifying the various stakeholders into three groups: core stakeholders, support stakeholders and peripheral stakeholders. We have identified various degrees of stakeholder involvement and integration, and have used this scale to develop a model of stakeholder integration in complex implementation projects in primary health care.

The findings contribute to the concept of organisational readiness [25] when it comes to innovation projects, and the data demonstrate that stakeholder inclusion at an early stage is critical to the success of the implementation. This also represents a contribution to the practical health context. An eHealth technology implementation project affects both structural and cultural aspects of the organisation, not least in relation to professions and power issues. The study contributes to underscoring that the stakeholders one may consider crucial, such as the IT department, are often overlooked. Conversely, stakeholders that might seem peripheral, like non-health care staff in the health care institution, should be involved throughout the process.

Furthermore, the research contributes to the field of private–public networks in services and shows that while the pro-active relationships between the health staff and the vendors may be novel for the public sector, they are nevertheless of vital importance. This is an area in rapid development and should receive considerable research efforts in the future. In addition, the eHealth project manager should aim to become a pro-active stakeholder that takes initiative in terms of building relationships within the frame of the implementation project.

We suggest the following be considered when embarking on eHealth technology initiatives in municipal health care: 1) identify important stakeholders in the early phase of the implementation process, and involve all key stakeholders to ensure the best possible outcome; 2) throughout the implementation process, the focus should be on creating arenas and routines for co-creation in the projects—this may ensure the innovation of products, services and systems that can benefit municipal health care and end users; 3) each stakeholder group has different needs for information and communication—these needs must be identified and routines must be in place to ensure they are met throughout the project.

### Issues for further research

The digitalisation of all levels of society is in rapid development, and this is also true for health care on the municipal level. The introduction and implementation of eHealth technology in community health care is under-researched, and there is a lack of both theoretical and practical contributions in this area. One suggestion for future research would be to further develop the model into a taxonomy of stakeholders in this setting and examine how these stakeholders can be managed. The power structures based on knowledge imbalance play a salient role in the context of the municipalities—the complexity of this context also requires increased research attention. Moreover, the challenge of integrating stakeholders within the municipality deserves attention, as innovation projects involve a number of stakeholders external to health care. This integration will also have potential for co-creation and learning. Finally, acknowledging the degree of involvement of informal caregivers (such as next of kin), future research should consider how the condition of the patient affects their involvement. Would the involvement differ if the patient did not have a cognitive condition but rather a physical one, and was thus able to advocate for themselves?

## Data Availability

The datasets generated and analysed during the current study are not publicly available due to privacy reasons, but are available from the corresponding author on reasonable request.

## Abbreviations

ERN: Etty R. Nilsen
IT support service: Information technology support service
IT department: Information technology department
KS: Karen Stendal
MKG: Monika K. Gullslett
SME: Small and medium sized enterprise
